# Surface Modification of Carbon Fiber-Polyetheretherketone Composite to Impart Bioactivity by Using Apatite Nuclei

**DOI:** 10.3390/ma14216691

**Published:** 2021-11-06

**Authors:** Yuya Yamane, Takeshi Yabutsuka, Yusuke Takaoka, Chihiro Ishizaki, Shigeomi Takai, Shunsuke Fujibayashi

**Affiliations:** 1 Department of Fundamental Energy Science, Graduate School of Energy Science, Kyoto University, Kyoto 606-8501, Japan; yamane.yuya.82a@st.kyoto-u.ac.jp (Y.Y.); ishizaki.chihiro.54z@st.kyoto-u.ac.jp (C.I.); stakai@energy.kyoto-u.ac.jp (S.T.); 2 Department of Orthopedic Surgery, Graduate School of Medicine, Kyoto University, Kyoto 606-8507, Japan; ytakaoka@kuhp.kyoto-u.ac.jp (Y.T.); shfuji@kuhp.kyoto-u.ac.jp (S.F.)

**Keywords:** carbon fiber-polyetheretherketone composite, apatite nuclei, apatite-forming ability, bioactivity, sulfuric acid treatment, oxygen plasma treatment

## Abstract

The authors aimed to impart the apatite-forming ability to 50 wt% carbon fiber-polyetheretherketone composite (50C-PEEK), which has more suitable mechanical properties as artificial bone materials than pure PEEK. First, the 50C-PEEK was treated with sulfuric acid in a short time to form pores on the surface. Second, the surface of the 50C-PEEK was treated with oxygen plasma to improve the hydrophilicity. Finally, fine particles of calcium phosphate, which the authors refer to as “apatite nuclei”, were precipitated on the surface of the 50C-PEEK by soaking in an aqueous solution containing multiple inorganic ions such as phosphate and calcium (modified-SBF) at pH 8.20, 25 °C. The 50C-PEEK without the modified-SBF treatment did not show the formation of apatitic phase even after immersion in simulated body fluid (SBF) for 7 days. The 50C-PEEK treated with the modified-SBF showed the formation of apatitic phase on the entire surface within 1 day in the SBF. The apatite nuclei-precipitated 50C-PEEK will be expected as a new artificial bone material with high bioactivity that is obtained without complicated fabrication processes.

## 1. Introduction

Polyetheretherketone (PEEK) is one of the super engineering plastic materials attracting attention because of its extreme toughness. The PEEK has already been in practical use as an implant for spinal fusion surgery. The PEEK has excellent tensile strength, flexural strength, fatigue resistance, impact resistance, wear resistance, and resistance to many chemicals over a wide temperature range. In addition, the bone-like elastic modulus and high specific strength of the PEEK are attractive properties for artificial bone materials. In general, the elastic modulus of human bone is approximately 20 GPa for cortical bone and 4–10 GPa for cancellous bone, respectively [1]. The elastic moduli of titanium alloys and stainless steels, which are widely used as implant materials in orthopedic fields such as intramedullary nails for the treatment of bone fractures, is approximately 100 GPa and 200 GPa, respectively, much higher than the natural bone [2,3]. However, too high elastic moduli of the artificial bone are possible to cause stress shielding, which is a phenomenon that the surrounding bone is absorbed because of the inactivation of bone remodeling due to the lack of average load to the bone. The stress shielding is possible to lead to problems such as loosening of the implant and re-fracturing if the implant needs to be replaced [4].

In contrast, the PEEK is more suitable for the artificial bone because the elastic modulus of pure PEEK is approximately 4 GPa, which is closer to that of human cortical bone than metallic materials [5]. In order to better utilize the advantages of PEEK, its composites with new material properties combined with various materials such as glass and carbon fiber have been developed. In addition to the advantages of PEEK described above, for example, 50 wt% carbon fiber–PEEK composite (50C-PEEK) shows higher tensile strength (129 MPa) and modulus (17.9 GPa) [6] more similar to the cortical bone than those of the pure PEEK (110 MPa, 4.34 GPa) [7]. For these reasons, PEEK and its composite have been expected to be a candidate for replacing conventional metal implants.

However, the pure PEEK and 50C-PEEK are still not utilized thoroughly in the clinical area despite their excellent mechanical properties. The disadvantage of both the pure PEEK and 50C-PEEK in clinical applications, especially as an artificial bone, is that they do not have bioactivity. The main inorganic component of the bone is non-stoichiometric hydroxyapatite, which has low crystallinity, deficiency of calcium, and inclusion of a minute amount of sodium and magnesium [8]. Bioactivity refers to the property of forming a layer of the apatite similar to the above on the surface of the materials in vivo and directly bonding with the bone through this apatite layer. Without this property, the material is coated with collagenous fibrous tissue and does not bond to living bone [9], which can cause problems in long-term fixation in vivo. For solving this problem, many methods to impart bioactivity to the PEEK have been reported [10]. For example, titanium oxide (TiO_2_) coating by the sol-gel process [11,12], sulfuric acid (H_2_SO_4_) treatment [13], H_2_SO_4_ and calcium chloride (CaCl_2_) treatment [14], and carbonate-rich hydroxyapatite coating by dynamic mineralization [15] have been reported. In addition, Prochor et al. reported that PEEK reinforced with 30% glass fiber showed hydroxyapatite formation [16]. However, these methods have problems such as complicated processing or taking several weeks for the formation of hydroxyapatite in simulated body fluid (SBF) [17], which is an aqueous solution with inorganic ion concentrations nearly equal to those of human plasma. Hence, a simple surface modification process to impart high bioactivity that can withstand clinical applications to PEEK in a short period of time has been required.

When the temperature and the pH of the SBF are increased, fine particles of amorphous calcium phosphate are precipitated. These particles cause the formation of apatite in a biomimetic environment, which we named ‘apatite nuclei’ [18,19]. In the recent studies, the authors developed a method to impart bioactivity to the pure PEEK and several types of ceramic fiber-PEEK composites (30 wt % carbon fiber-PEEK composite, 30 wt % glass fiber-PEEK composite, and 10 wt % carbon nanotube-PEEK composite) [20,21,22]. In this method, H_2_SO_4_ treatment for the PEEKs was carried out as a pore formation process, and an oxygen plasma treatment was subsequently carried out for improvement of their hydrophilicity. Next, the pH of the SBF was raised to 8.40, 25.0 °C by dissolving tris(hydroxymethyl)aminomethane ((CH_2_OH)_3_CNH_2_) and then the pores-formed PEEKs were soaked in the SBF. Finally, the temperature of the SBF was raised for the acceleration of calcium phosphate precipitation. As a result, the apatite nuclei were precipitated on the surface and in the pores of the PEEKs, and the apatite-forming ability was imparted to the surface of the PEEKs. This method does not require a complicated surface modification process, and it has been confirmed that the apatitic phase was formed within 1 day in the conventional SBF [20,21,22]. In addition, the authors have already reported that the pure PEEK modified with the apatite nuclei showed good bone-bonding ability in rabbit tibia [23]. In this study, the authors aimed to impart bioactivity to not only the pure PEEK but also the 50C-PEEK with suitable mechanical strength and modulus for the artificial bone by the precipitation of the apatite nuclei on their surfaces.

In the previous studies, the authors imparted the apatite-forming ability to yttria-stabilized zirconia (YSZ) and zirconium (Zr) alloy, which are bioinert materials, by the combination of pores formation and the above apatite nuclei precipitation [24,25]. In the above studies, the authors applied a modified-SBF, which is an aqueous solution prepared by dissolving only CaCl_2_, di-potassium hydrogen phosphate trihydrate (K_2_HPO_4_·3H_2_O), and magnesium chloride hexahydrate (MgCl_2_·6H_2_O) among inorganic chemical reagents used for the conventional SBF except pH regulators in pure water instead the conventional SBF was used to precipitate apatite nuclei on the YSZ and the Zr alloy. Several types of physiological biomineralization media, such as the SBF, Ringer’s solution, Hank’s solution, Earle’s solution, etc., have already been proposed [26]. On the other hand, it has been reported that the presence of 0.15 mol·dm^−3^ sodium chloride (NaCl) in the reaction solution remarkably decreases the crystallization rate of apatitic calcium phosphate [26,27]. The characteristic point of the modified-SBF in comparison with other types of media is that the NaCl was not dissolved as a solute in the preparation process. By using the modified-SBF, the apatite nuclei precipitation showed higher reproducibility in the cases of the YSZ and the Zr alloy compared to the case of the conventional SBF [24,25]. Therefore, the modified-SBF was used for the purpose of promoting and improving the reproducibility of the apatite nuclei precipitation on the PEEKs in this study based on the experience in the above previous study. The authors evaluated the effect on the apatite nuclei precipitation on the pure PEEK and the 50C-PEEK and the apatite-forming ability in the SBF in this study.

## 2. Materials and Methods

### 2.1. Preparation of SBF and modified-SBF

Table 1 shows the inorganic ion concentrations in the SBF, the modified-SBF, and human blood plasma. The SBF was prepared by dissolving reagent grade NaCl (FUJIFILM Wako Pure Chemical, Osaka, Japan), sodium hydrogen carbonate (NaHCO_3_) (Hayashi Pure Chemical, Osaka, Japan), potassium chloride (KCl) (Hayashi Pure Chemical), K_2_HPO_4_·3H_2_O (Nacalai Tesque, Kyoto, Japan), MgCl_2_·6H_2_O (Hayashi Pure Chemical), 1 mol·dm^−3^ hydrochloric acid (HCl) (Hayashi Pure Chemical), CaCl_2_ (Hayashi Pure Chemical), and sodium sulfate (Na_2_SO_4_) (Hayashi Pure Chemical) in 1 dm^3^ of distilled water in the amounts shown in Table 2 in order. Subsequently, the solution was adjusted at pH 7.40, 36.5 °C using 1 mol·dm^−3^ HCl and (CH_2_OH)_3_CNH_2_ (Hayashi Pure Chemical). The modified-SBF was prepared by dissolving the same amount of K_2_HPO_4_·3H_2_O, MgCl_2_·6H_2_O, and 1 mol·dm^−3^ HCl in 1 dm^3^ of distilled water and CaCl_2_ with the case of the SBF and adjusted at pH 8.20, 25.0 °C using (CH_2_OH)_3_CNH_2_. The SBF was prepared for the evaluation of the apatite-forming ability and the modified-SBF for the apatite nuclei precipitation for the impartation of bioactivity to the PEEKs.

The reaction solution for the apatite nuclei precipitation was required to prevent their precipitation before soaking the objective substrate, that is, before raising the temperature, to achieve uniform precipitation of the apatite nuclei on the entire surface of the substrate. As described in the Introduction, the SBF adjusted at pH 8.40, 25.0 °C was used for apatite nuclei precipitation in the previous study [20,21,22]. However, the pH of the modified-SBF was set to 8.20 because the apatite nuclei were precipitated in the modified-SBF before soaking the substrate instead of after if the pH was raised to higher than around 8.20.

### 2.2. Substrates

Plates of the PEEK (Ketron^®^ 1000 PEEK, 129 MPa in tensile strength, 4.34 GPa in tensile modulus, Mitsubishi Chemical Advanced Materials, Tokyo, Japan) and the 50C-PEEK (TECAPEEK^®^ CM XP111 BLACK, carbon fibers: 50 wt %, 110 MPa in tensile strength, 17.9 GPa in tensile modulus, Ensinger, Nufringen, Germany) with 15 mm × 10 mm × 2 mm in size were used as substrates. The surfaces of the PEEKs were polished using #400 and subsequently #1200 silicon carbide (SiC) abrasive papers and then washed ultrasonically in acetone, ethanol, and distilled water each for 10 min. Thus-treated PEEKs were air-dried at room temperature. The obtained samples are donated as ’Sample N’ hereafter.

### 2.3. Surface Modification

#### 2.3.1. H_2_SO_4_ Treatment

In order to form pores on the surface of the Sample N, the Sample N was soaked in 40 cm^3^ of 95 wt % H_2_SO_4_ (FUJIFILM Wako Pure Chemical) for 4 s in total. Thus, treated PEEKs were washed ultrasonically in distilled water for 10 min. The obtained samples are denoted as ‘Sample S’ hereafter.

#### 2.3.2. Oxygen Plasma Treatment

For the impartation of hydrophilicity to the surface of the PEEKs, both surfaces of Sample S were treated with oxygen plasma at 200 W for 4 min using glow discharge equipment (Model BP-1, Samco, Kyoto, Japan). The obtained samples are denoted as ‘Sample SP’ hereafter.

#### 2.3.3. Modified-SBF Treatment

In order to precipitate the apatite nuclei on the surface of the Sample SP, the Sample SP was immersed in the modified-SBF just after the oxygen plasma treatment. Then, the modified-SBF was kept in an incubator held at 70.0 °C for 24 h. After the immersion in the modified-SBF, the samples were washed with distilled water for 30 s and air-dried. The obtained samples are denoted as ‘Sample SPA’ hereafter.

### 2.4. Evaluation of the Apatite-Forming Ability

For the evaluation of the apatite-forming ability, each sample described above (Samples N, S, SP, and SPA) was immersed in the SBF at pH 7.40, 36.5 °C for 1 day, 4 days, and 7 days. After the immersion in the SBF, the samples were washed with distilled water and air-dried. All the prepared samples were stored in a plastic case under normal temperature and pressure until the analyses described below.

### 2.5. Analyses

The surface of each sample was analyzed with field emission scanning electron microscopy (SEM; SU6600, Hitachi High-Technologies, Tokyo, Japan), energy dispersive X-ray spectroscopy (EDX; XFlash^®^ 5010, Bruker, Billerica, M.A.), X-ray photoelectron spectroscopy (XPS; JPS-9010TRX, JEOL, Tokyo, Japan) using Mg-Kα radiation at 10 kV and 10 mA in tube voltage and current, thin-film X-ray diffraction (TF-XRD; X’Pert P, PANalytical, Almelo, Netherland) by the parallel beam method using Cu-Kα radiation at 45 kV and 40 mA in tube voltage and current, and Fourier transform infrared spectroscopy (FT-IR; FT/IR-4700, JASCO, Tokyo, Japan) at 4 cm^−1^ resolution with the attenuated total reflection method using a diamond prism. Before the SEM and EDX observation, gold was coated on the substrates by a sputtering method. In the XPS analyses, Ca/P atomic ratio was estimated based on the obtained spectra using 1 sample for each condition. Water contact angles were measured with a contact angle meter (Smart Contact PRO 100 Ⅱ, Excimer, Yokohama, Japan) using 5 samples for each condition. For the evaluation of significance, a one-way analysis of variance (ANOVA) followed by Tukey’s multiple comparison test was applied to the water contact angles among 4 groups (Samples N, S, SP, and SPA). The above statistical analyses were performed using OriginPro (Version 2021, OriginLab Corporation, Northampton, MA, USA).

## 3. Results

### 3.1. Materials Analyses

#### 3.1.1. Changes in Surface Morphology

**Figure 1:**
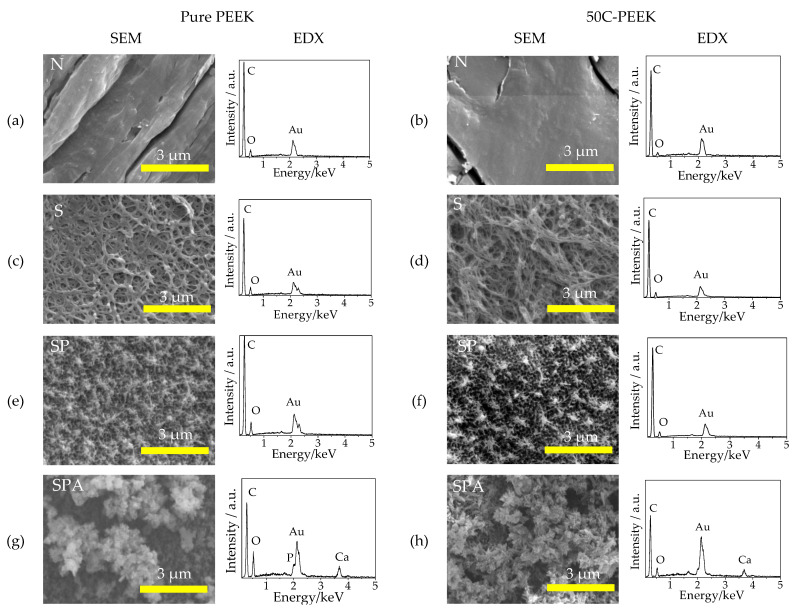
SEM micrographs and EDX profiles on the surfaces of Samples (**a**,**b**) N: untreated PEEKs, (**c**,**d**) S: sulfuric acid-treated PEEKs, (**e**,**f**) SP: sulfuric acid + oxygen plasma-treated PEEKs, and (**g**,**h**) SPA: sulfuric acid + oxygen plasma + modified-SBF-treated PEEKs for (**a**,**c**,**e**,**g**) pure PEEK and (**b**,**d**,**f**,**h**) 50C-PEEK.

Figure 1a,b shows the SEM micrographs and the EDX profiles of the surfaces of Sample N, which was the untreated pure PEEK and the 50C-PEEK. Because of the polishing process, the surface of the samples was smooth. In the EDX, the peaks of C, O, and Au were observed. The C and O were derived from the PEEKs and the Au from gold coating for the pre-treatment for the observation.

Figure 1c,d shows the SEM micrographs and the EDX profiles of the surfaces of Sample S, which was the pure PEEK and the 50C-PEEK treated with H_2_SO_4_. The surface of the PEEKs substrate was dissolved in H_2_SO_4_ and the pores with approximately 500 nm in diameter were formed. The EDX profiles showed similar results to Sample N.

Figure 1e,f shows the SEM micrographs and the EDX profiles of the surfaces of Sample SP, which was the pure PEEK and the 50C-PEEK treated with H_2_SO_4_ and subsequently oxygen plasma. After irradiating oxygen plasma, it was confirmed that the size of the pores became fine and that the surface became a bit rougher than that of Sample S.

Figure 1g,h shows the SEM micrographs and the EDX profiles of the surfaces of Sample SPA, which was the pure PEEK and the 50C-PEEK treated with H_2_SO_4_, oxygen plasma, and subsequently the modified-SBF. In the SEM micrographs, the surface of the PEEKs was entirely covered with particles approximately 0.5 µm in diameter. Furthermore, the peaks of Ca and P were newly observed in the EDX profiles. Given these results, it is considered that the fine particles of calcium phosphate were formed by the modified-SBF treatment.

#### 3.1.2. Changes in Functional Groups

**Figure 2:**
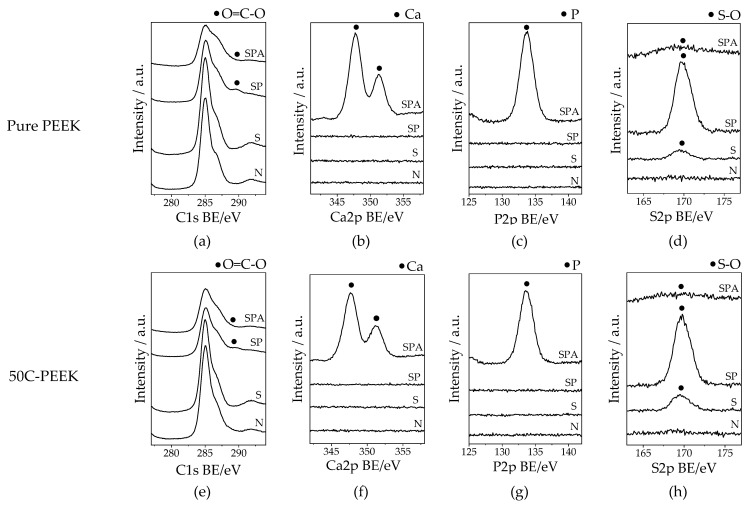
XPS narrow spectra around binding energy of C1s, Ca2p, P2p, and S2p on the surfaces of (**a**–**d**) pure PEEK and (**e**–**h**) 50C-PEEK.

Figure 2 shows the XPS profiles on the surfaces of Samples N, S, SP, and SPA of the pure PEEK and the 50C-PEEK. For both the PEEK and the 50C-PEEK, similar changes were observed in all the treatments. For Sample N, no peaks were observed in S2p, P2p, and Ca2p, and a strong peak was observed in C1s. For Sample S, there was the peak derived from S-O in S2p. For Sample SP, the intensity of the peak derived from S-O was larger than that of Sample S, and the peak derived from O=C-O was newly detected in C1s. For Sample SPA, the peaks of Ca and P were detected in Ca2p and P2p, and the intensity of the peaks derived from S-O and O=C-O were smaller than from Sample SP. These results indicated that the sulfonic groups and carboxyl groups were introduced by the H_2_SO_4_ treatment and the subsequent oxygen plasma treatment, respectively, and the apatite nuclei containing Ca and P covered the surface of the PEEKs by the modified-SBF treatment. From the results of the quantitative analyses based on the spectra shown in Figure 2, the Ca/P atomic ratios of the precipitated apatite nuclei were 1.20 for the pure PEEK and 1.17 for the 50C-PEEK, respectively. It was shown that the Ca/P atomic ratios of the apatite nuclei on both the PEEKs were almost at a comparative level and clearly lower than 1.67, which is that of stoichiometric hydroxyapatite.

#### 3.1.3. Hydrophilicity

**Figure 3:**
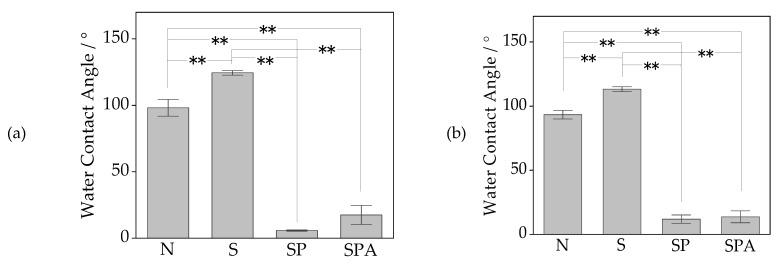
Water contact angles on the surfaces of Samples N (untreated PEEKs), S (sulfuric acid-treated PEEKs), SP (sulfuric acid + oxygen plasma-treated PEEKs), and SPA (sulfuric acid + oxygen plasma + modified-SBF-treated PEEKs) for (**a**) pure PEEK and (**b**) 50C-PEEK. The symbol ‘**’ indicates *p* < 0.01 and no symbol *p* > 0.05 by a one-way ANOVA, followed by Tukey’s test.

Figure 3 shows the water contact angles on the surfaces of the Samples N, S, SP, and SPA for the pure PEEK and the 50C-PEEK. For both the pure PEEK and the 50C-PEEK, remarkable differences were not shown. The average values and standard deviations of the WCA of the Samples N, S, SP and SPA for the pure PEEK was 98.2 ± 4.2°, 124.4 ± 1.2°, 5.7 ± 0.4°, and 17.4 ± 4.8° and those of the 50C-PEEK were 93.3 ± 2.3°, 113.2 ± 1.3°, 11.9 ± 2.2°, and 13.7 ± 3.1°, respectively. All of them were computed from five samples.

The surfaces of Sample N showed hydrophobicity, and Sample S was more hydrophobic than Sample N. On the other hand, the surface of Samples SP and SPA showed hydrophilicity. The statistically significant difference (*p* < 0.01) was shown in all the comparisons except between SP and SPA. These results revealed that the hydrophilicity of the surface of the PEEKs drastically increased by the oxygen plasma treatment, and the obtained hydrophilicity was almost kept even after the modified-SBF treatment. 

### 3.2. Evaluation of Apatite-Forming Ability

**Figure 4:**
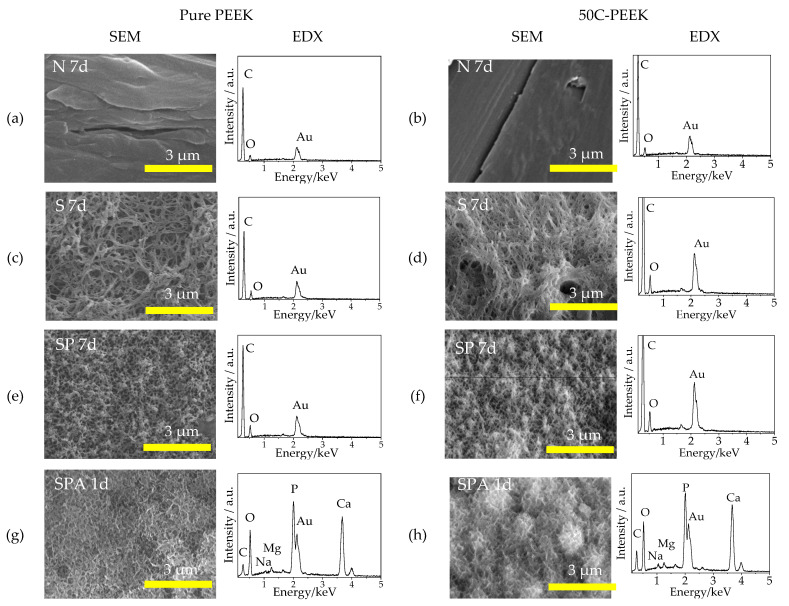
SEM micrographs and EDX profiles of the surfaces of Samples (**a**,**b**) N: untreated PEEKs after immersion in SBF for 7 days, (**c**,**d**) S: sulfuric acid-treated PEEKs after immersion in SBF for 7 days, (**e**,**f**) SP: sulfuric acid + oxygen plasma-treated PEEKs after immersion in SBF for 7 days, and (**g**,**h**) SPA: sulfuric acid + oxygen plasma + modified-SBF-treated PEEKs after immersion in SBF for 1 day for (**a**,**c**,**e**,**g**) pure PEEK and (**b**,**d**,**f**,**h**) 50C-PEEK.

Figure 4 shows the SEM micrographs and the EDX profiles of Sample N after the immersion in the SBF for 7 days, Sample S for 7 days, Sample SP for 7 days, and Sample SPA for 1 day. In the SEM micrographs, it was confirmed that the apatitic phase did not form on the surfaces of Samples N, S, and SP even after the immersion in SBF for 7 days. On the other hand, flake-like crystallites characteristic to apatite formed in the SBF were observed in the surfaces of Sample SPA after immersion for 1 day. In the EDX profiles, the intensified peaks of Ca and P and slight peaks of Na and Mg were detected from Sample SPA after immersion for 1 day.

**Figure 5:**
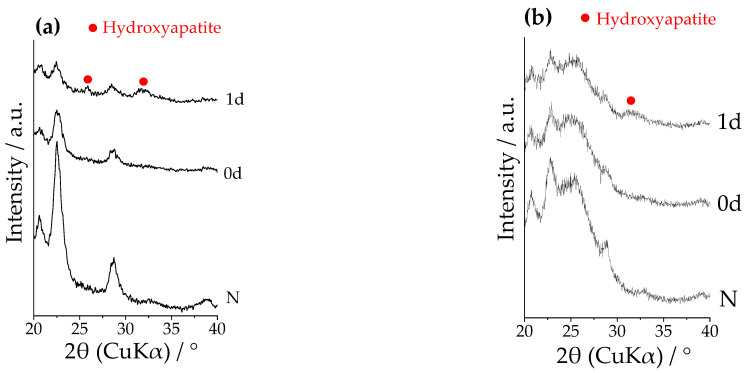
TF-XRD profiles on the surfaces of Samples N: untreated PEEKs (denoted as “N”) and SPA: sulfuric acid + oxygen plasma + modified-SBF-treated PEEKs before (denoted as “0d”) and after immersion in SBF for 1 day (denoted as “1d”) for (**a**) pure PEEK and (**b**) 50C-PEEK.

Figure 5 shows the TF-XRD profiles of Samples N and SPA before and after the immersion in SBF. The peaks attributed to hydroxyapatite were detected around 26° and 32° on the surface of Sample SPA for the pure PEEK after the immersion in SBF for 1 day. In that for the 50C-PEEK, a peak around 32° was detected, but the peak around 26° was not distinguishable. It was considered that this was because the peak around 25–26° derived from the 50C-PEEK substrate overlapped with the peak around 26° attributed to hydroxyapatite.

**Figure 6:**
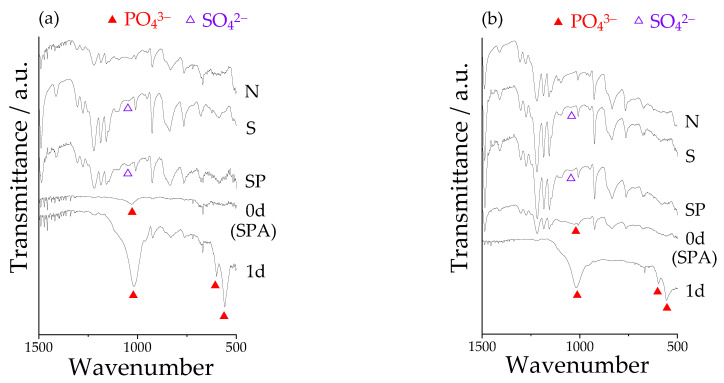
FT-IR spectra on the surface of Samples N: untreated PEEKs, S: sulfuric acid-treated PEEKs, SP: sulfuric acid + oxygen plasma-treated PEEKs, and SPA: sulfuric acid + oxygen plasma + modified-SBF-treated PEEKs before (denoted as “0d”) and after immersion in SBF for 1 day (denoted as “1d”) for (**a**) pure PEEK and (**b**) 50C-PEEK.

Figure 6 shows the FT-IR spectra of Samples N, S, SP, and SPA before and after immersion in SBF. After the H_2_SO_4_ and subsequent oxygen plasma treatment, the slight absorption band attributed to O=S=O, which indicated the existence of the sulfonic groups, was observed around 1050 cm^−1^. After the modified-SBF treatment, the absorption band attributed to P=O, which indicated the existence of the phosphate groups, was observed around 1000 cm^−1^. After the immersion in SBF, furthermore, the large absorption bands of P=O were observed around 1000 cm^−1^, 600 cm^−1^, and 550 cm^−1^, respectively. These results suggested that fine particles observed in Figure 1g,h, that is, “apatite nuclei”, took up the phosphate groups from the SBF and caused crystal growth of the apatitic phase in the SBF.

These results indicated that the pure PEEK and the 50C-PEEK treated with H_2_SO_4_ for the formation of the pores, oxygen plasma for the introduction of the hydrophilicity, and subsequently the modified-SBF for the precipitation of the apatite nuclei showed formation ability of the apatitic phase within 1 day in the SBF.

## 4. Discussion

In this study, the authors firstly treated the pure PEEK and the 50C-PEEK with H_2_SO_4_. As already reported, pores formation on the surfaces of various types of PEEKs was observed in SEM micrographs [20,21,22]. Moreover, a similar phenomenon was observed even in the case of the 50C-PEEK in the present study. Hence, the reaction to form pores was expected to be caused by the PEEK matrix regardless of the presence of carbon fibers. This phenomenon was thought to be caused by the reaction between the aromatic groups of the aromatic compounds and the sulfonic groups of H_2_SO_4_ as follows [28].

C_6_H_6_ + H_2_SO_4_ ↔ C_6_H_5_SO_3_H + H_2_O (1)

The results of the XPS analyses after the H_2_SO_4_ treatment, in which sulfonic groups were introduced to the surfaces of the PEEKs, were also consistent with this reaction. Even after the oxygen plasma treatment, the morphology and the functional groups of the surfaces of the substrate changed both the pure PEEK and the 50C-PEEK. The introduction of the carboxy groups was one of the important changes by the oxygen plasma treatment. As described in Comyn’s paper [29], such an introduction of the functional groups possibly indicated that oxygen attacked carbonyl groups, and the -Ph-COOH groups were newly produced. In addition, the XPS analyses showed an increase in the peak intensity of sulfonic groups due to the oxygen plasma treatment. This result might have been caused by a different reason than the introduction of the sulfonic groups by the H_2_SO_4_ treatment. In other words, the surface was roughened by the heat of the oxygen plasma, and the sulfonic groups generated inside the substrate by the penetration of the H_2_SO_4_ may have been exposed on the surface. From the water contact angle measurements, the hydrophilicity was greatly improved by the oxygen plasma treatment. According to the Wenzel equation [30], the contact angle was the product of the chemical properties and the surface roughness factor, and it was stated that the increase in surface roughness makes hydrophobic surfaces more hydrophobic and hydrophilic surfaces more hydrophilic. The results of this study on the water contact angle measurements are likely explained as follows. In the H_2_SO_4_ treatment, there seemed to be a significant effect of increased hydrophobicity due to the roughening of the originally hydrophobic PEEKs surface, resulting in an increase in the contact angles despite the introduction of the hydrophilic sulfonic groups. In the case of the oxygen plasma treatment, the effects of increased sulfonic groups and the introduction of the carboxy groups seemed to exceed the effects of the hydrophobicity of the substrate itself, and combined with the effects of the surface roughening, a significant decrease in the water contact angles was observed. The increased hydrophilicity had a positive effect on the subsequent modified-SBF treatment and the SBF immersion tests in terms of better penetration of the liquid into the PEEKs substrate.

After the modified-SBF treatment, fine particles of calcium phosphate, which the author named “apatite nuclei”, were deposited on the entire surfaces of the PEEKs substrate. In our previous studies, the precipitation of the apatite nuclei on PEEK by immersion in the SBF with increased pH [21] had been confirmed. When the material was changed to the 50C-PEEK and treated with the same composition of SBF, however, the apatite nuclei precipitation was not stably observed, probably because of the large number of carbon fibers present. Such difficulty in the apatite nuclei precipitation was observed in the case of the YSZ and the Zr alloy in our previous study [24,25]. In this study, the authors confirmed more highly reproducible precipitation of the apatite nuclei on the pure PEEK and the 50C-PEEK by the modified-SBF treatment similar to the case of the YSZ and the Zr alloy. Therefore, it was suggested that the modified-SBF had the ability to impart the apatite-forming ability to materials that are relatively difficult to obtain apatite-forming ability using the conventional SBF by a simpler method. In addition, the simpler ion composition of the modified-SBF is expected to control the reproducibility of material properties simply and precisely.

Generally, hydroxyapatite formation in an aqueous solution can be described as shown below.

10Ca^2+^ + 6PO_4_^3−^ + 2OH^−^ ↔ Ca_10_(PO_4_)_6_(OH)_2_ (2)

As the pH value of the aqueous solution increases, the increase in hydroxy ions and the formation of hydroxyapatite proceed in terms of chemical equilibrium. Furthermore, in this study, the treatment was performed under temperatures (70.0 °C) much higher than the physiological temperature (36.5 °C). Hence, it is considered that the formation reaction of calcium phosphate was accelerated.

The Ca/P atomic ratio of the apatite nuclei obtained by the XPS was approximately 1.2 for both the PEEKs. On the other hand, the authors have reported that the Ca/P atomic ratio of the apatite nuclei precipitated on the surface of the pure PEEK using the conventional SBF was 1.62 [23]. It is possible that such a difference in the Ca/P atomic ratio was led by the effect of the different ionic strength and supersaturation with respect to calcium phosphates because of the difference in the inorganic ion composition between the conventional SBF and the modified-SBF.

The results of the SBF tests showed that the formation of the apatitic phase was observed on the surfaces of Sample SPA within 1 day in the SBF, while it was not observed in Samples N, S, and SP even after 7 days. Hence, the apatite nuclei precipitation was an essential factor in imparting the apatite-forming ability to the PEEKs in the presented experimental system. Considering the results of the EDX and TF-XRD of Sample SPA after the immersion in SBF, the apatitic phase included sodium and magnesium, possessed low crystallinity, and seemed to be similar to the characteristics of apatite in the living bone. Zhao et al. reported that PEEK treated with H_2_SO_4_ showed apatite formation within 28 days in SBF [13]. In addition, Miyazaki et al. reported that PEEK and 30% carbon–PEEK composite treated with sulfuric acid and subsequent calcium chloride also showed apatite formation within 14 days in SBF [14]. Compared to these previous studies, the method of imparting bioactivity by the apatite nuclei precipitation using modified-SBF treatment was an effective method in terms of a short induction period for the apatitic phase formation. In addition, it was clarified that such high formation ability of the apatitic phase was shown even the carbon fibers contained at the weight ratio of 50 wt% in the PEEK matrix. As already mentioned above, in addition, the authors have reported that the modified-SBF treatment was effective in imparting apatite-forming ability not only for PEEKs but also bioinert metals and ceramics [24,25]. From these findings, it was suggested that the presented bioactivity treatment, that is, the apatite nuclei precipitation using the modified-SBF-treatment, possessed a wide range of materials selectivity.

On the other hand, the authors acknowledge the following limitations in this study. The thickness of the layer of apatite nuclei on the PEEKs has not been clarified yet. In our previous study, the authors clarified that the thickness of the layer of the apatite nuclei on the surface of the H_2_SO_4_ and subsequent oxygen plasma-treated pure PEEK precipitated using the SBF instead of the modified-SBF was less than 5 μm [23]. It is considered that the thickness of the layer of the apatite nuclei was related to the depth of the porous structures on the surface of the substrate. From this point, it is predicted that the thickness in this study was comparative level because the experimental conditions before the apatite nuclei precipitation were the same except the point using the 50C-PEEK as substrates. In addition, the authors have not evaluated the bone formation in vivo, gene expression, alkali phosphatase activity, and cell viability for the PEEKs treated with the presented conditions yet. These points will be clarified in future studies.

## 5. Conclusions

The authors treated the pure PEEK and the 50C-PEEK by the pore formation with the H_2_SO_4_ treatment, the hydrophilic reaction with the oxygen plasma treatment, and the subsequent deposition of the apatite nuclei with the modified-SBF treatment. In each treatment, there was no significant difference between the pure PEEK and the 50C-PEEK, and the 50C-PEEK after all the treatments was found to form an apatitic phase within 1 day in the SBF that mimicked the biological environment, similar to other types of PEEK composites reported so far. Therefore, the 50C-PEEK and this kind of PEEK composite with better mechanical properties than the pure PEEK will be increasingly studied for biomedical applications in the future.

## 6. Patents

Yao, T.; Hibino, M.; Yabutsuka, T. Method for Producing Bioactive Composites. U.S. Patent 8512732, 20 August 2013, Japanese Patent 5252399, 26 April 2013.

## Figures and Tables

**Table 1:** Inorganic ion concentrations in SBF [17], modified-SBF, and human blood plasma [17].

Ion	Ion Concentration/mmol∙dm^−3^		
	SBF	Modified-SBF	Blood Plasma
Na^+^	142.0	-	142.0
K^+^	5.0	2.0	5.0
Mg^2+^	2.5	2.5	2.5
Ca^2+^	1.5	1.5	1.5
Cl^−^	147.8	8.35	103.0
HCO_3_^−^	4.2	-	27.0
HPO_4_^2−^	1.0	1.0	1.0
SO_4_^2−^	0.5	-	0.5

**Table 2:** Amount or volume of dissolved reagents in the preparation of 1 dm^3^ of SBF [17] and modified-SBF.

Reagents	Amount or Volume of the Reagents	
	SBF	Modified-SBF
NaCl	7.996 g	-
NaHCO_3_	0.350 g	-
KCl	0.224 g	-
K_2_HPO_4_∙3H_2_O	0.228 g	0.228 g
MgCl_2_·6H_2_O	0.350 g	0.350 g
1 mol·dm^−3^ HCl	35 cm^3^	35 cm^3^
CaCl_2_	0.278 g	0.278 g
Na_2_SO_4_	0.071 g	-

## Data Availability

The data presented in this study are available upon request from the corresponding author.
